# Effect of Photoactivation by Ultraviolet Light on Bond Strength of Composite Veneer on Stainless Steel Crowns—An *In Vitro* Study

**DOI:** 10.5005/jp-journals-10005-1593

**Published:** 2019

**Authors:** Aakash Sharma, Sadanand Kulkarni, Kattebelaguli VN Swamy

**Affiliations:** 1 Department of Pediatric and Preventive Dentistry, Sri Aurobindo College of Dentistry, Indore, Madhya Pradesh, India; 2,3 Department of Pedodontics and Preventive Dentistry, Sri Aurobindo College of Dentistry, Indore, Madhya Pradesh, India

**Keywords:** Composite resin, Early childhood caries, Shear bond strength, Stainless steel crowns (SSC), UV irradiation

## Abstract

**Aim:**

The aim of this study is to determine the effect of ultraviolet irradiation on the bond strength of composite veneer adhered to the SSCs.

**Materials and methods:**

Seventy anterior typhodont teeth (API, New Delhi, India) were randomly divided into two groups (*N* = 35/group) to be crowned with 70 maxillary left central incisor SSCs, size no. 3 (3MESPE, St. Paul, USA). The crowns were adjusted and cemented with the glass ionomer cement (type I, Ivoclar Vivadent, New York, USA). The labial surfaces of the experimental group were exposed to UV irradiation for 80 minutes using the UV chamber (Easy UV Chamber, India) with 2 UV lamps that produced 30 W of power to induce photoactivation. Standardized composite blocks (Ivoclar Vivadent, Gurgaon, India) of 4 × 4 × 1 mm were fabricated using Teflon molds and light cured for 60 seconds. The samples were fixed in the acrylic resin (NicTone62),with a label bearing the number of each sample. The samples were stored in a dry medium for 24 hours and tested using a universal testing machine.

**Results:**

The mean shear bond strength in the non-UV group was 26.03 ± 9.42 MPa, while in the UV group, it was 35.10 ± 14.80 MPa. Thus, there was a statistically significant difference in the mean value of the shear bond strength between the non-UV and UV groups. The shear bond strength in the UV group is much higher as compared with the non-UV group.

**Conclusion:**

Based on this study's results, the following conclusion can be made: ultraviolet irradiation of pediatric stainless steel crowns was found to significantly increase the shear bond strength of composite resin.

**Clinical significance:**

UV irradiation could provide suitable adhesion of composite resins to stainless SSCs, leading to in-office veneering of SSCs.

**How to cite this article:**

Sharma A, Kulkarni S, *et al.* Effect of Photoactivation by Ultraviolet Light on Bond Strength of Composite Veneer on Stainless Steel Crowns—An In *Vitro Study*. Int J Clin Pediatr Dent 2019;12(1):50–52.

## INTRODUCTION

Dental caries is a common dental disease that affects all populations, regardless of age, race, or gender.^1^ Children represent a high-risk group to develop caries^2,3^ known as early childhood caries (ECC), before they are 6 years old.^4,5^ Loss of primary teeth is common sequelae of ECC altering the development of occlusion, phonation, permanent tooth eruption, and esthetic appearance. Restoration of a primary incisor tooth is a challenge to dentists due to severe tooth destruction; operative dentistry could solve these problems, especially for teeth restored with stainless steel crowns (SSCs), veneer crowns, polycarbonate crowns, and strip crowns.^6^

Since the introduction of SSCs by Humphrey in the 1950s, the SSCs have been used in multisurface carious teeth. Stainless steel crowns are retentive, easy to place, and durable, but the metallic appearance may be esthetically displeasing to both parent and child.^7^ Open-faced stainless steel crowns with a resin window give improved esthetics over traditional stainless steel crowns. Polycarbonate crowns while having improved esthetics are considered to be more difficult to place, have poorer retention, and are prone to excessive wear. Acid-etched composite resin crowns or “strip crowns” are the most esthetic of the crowns showing improved retention and better wear resistance than polycarbonate crowns; however, they are more technically sensitive to place compared to prefabricated crowns.^8,9^

An important addition to the armamentarium of esthetic anterior primary tooth restorations is the veneered stainless steel crown.^10^ These crowns are available with a variety of facing materials such as composite resin or thermoplastic resin bonded to the stainless steel crown. Esthetic veneers are retained on the stainless steel crown using a variety of mechanical and chemical bonding approaches.^11^ An advantage of these restorations includes enhanced esthetics and retention that should be similar to the traditional stainless steel crowns.

In-office veneering of crowns has been reported and involves bonding resin composite and compomer directly to the SSCs.^12^ The photocatalysis effect by ultraviolet (UV) irradiation of titanium oxide (TiO) has been reported to enhance the attachment of different cell lines, thus, increasing the shear bond strength of the segmented polyurethane.^13–15^ Thus, UV irradiation could be a suitable application to increase the retention of veneer facing over stainless steel crowns. Hence, the purpose of this study was to determine the effects of photoactivation by UV light on bond strength of composite veneer on stainless steel crowns.

## MATERIALS AND METHODS

**Fig. 1:**
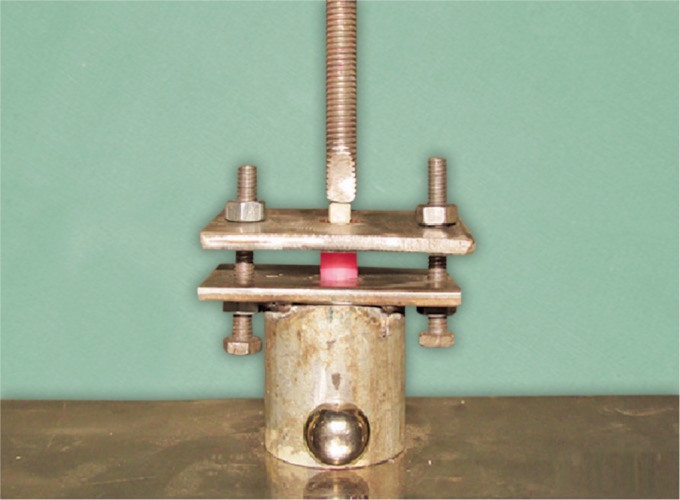
Testing of samples using a universal testing machine in the shear mode

Seventy anterior typhodont teeth (API, New Delhi, India) were randomly divided into two groups (*N* = 35/group) to be crowned with 70 maxillary left central incisor SSCs, size no. 3 (3M ESPE, St. Paul, USA). The crowns were adjusted and cemented with the glass ionomer cement (type I, Ivoclar Vivadent, New York, USA). To improve the retention of the facing, the vestibular surface of all crowns was roughened with a green mounted stone (Rhino, MDC, Cuadalajara) at a low speed for 5 seconds. The labial surfaces of the experimental group were exposed to UV irradiation for 80 minutes using the UV chamber (Easy UV Chamber, India) with 2 UV lamps that produced 30 W of power to induce photoactivation. A thin layer of metal composite bond (SR linking bonding system, Ivoclar Vivadent, Gurgaon, India) was applied to the crown surfaces of all teeth with a small brush and dried for 3 minutes. Two layers of opaquer (Opaquer A2, Adoro Shofu Dental Corporation, San Marcos, USA) were placed over the surface and light cured (Optilight, Gnatus, Ribeirao Preto, Sao Paulo, Brazil) for 60 seconds. Standardized composite blocks (Ivoclar Vivadent, Gurgaon, India) of 4 × 4 × 1 mm were fabricated using Teflon molds and light cured for 60 seconds. Each composite block was bonded over the crowns with the composite and light cured for 60 seconds. The samples were fixed in the acrylic resin (NicTone 62), with a label bearing the number of each sample. A mounting jig was used to align each tooth's labial surface. The samples were stored in a dry medium for 24 hours and tested using a universal testing machine (AGS-X, Shimadzu, Kyoto, Japan) in the shear mode (Fig. 1). The force was applied at the interface of the composite resin block and the crown. The bond strength was measured at a crosshead speed of 1 mm/minute, and the force of debonding will be recorded in Newtons (N) and converted into megapascals (MPa). All data were examined and tested for normality distribution. The mean values and standard deviations were estimated. Student's *t* test was used to compare the shear bond strength between the groups. To find out the statistical difference between the two mean values, the Students unpaired “*t*” was applied. The value of “*t*” obtained was −3.02 with a degree of freedom of 66; the *p* value obtained was <0.05, which is statistically significant.

**Table 1:** Mean shear bond strength in both the groups (*N* = 68)

*Group*	*Shear bond strength (MPa) mean ± SD*	*“*t*” value*	*Df*	*p value*
Non-UV group	26.03 ± 9.42	−3.02	66	*p* = 0.004*
UV group	35.10 ± 14.80			

Students unpaired “*t*” test

* Statistically highly significant

## RESULTS

Table 1 shows the mean shear bond strength in both non-UV and UV groups. The mean shear bond strength in the non-UV group was 26.03 ± 9.42 MPa, while in the UV group, it was 35.10 ± 14.80 MPa.

Thus, there was a statistically significant difference in the mean value of the shear bond strength between the non-UV and UV groups. The shear bond strength in the UV group is much higher as compared with the non-UV group.

## DISCUSSION

For restoring multisurface carious primary teeth, full coverage restorations are often the reliable option. Since anterior and posterior teeth are restored with stainless steel crowns, the need for an esthetic alternative to stainless steel crowns for anterior teeth has been recognized. As parental demand for esthetics for anterior teeth is rising day by day, dentists are forced to look at more esthetic options such as preveneered or open-faced crowns. There are drawbacks with preveneered stainless steel crowns such as difficulties in shade matching, limited ability to crimp the crown, and tendencies of fracture of the veneered surfaces. Conversely, the technique described in this study could be an alternative to solve the problem of antiesthetic appearance of SSCs.

It has been found that mechanical modifications improve the bond strength between composites and stainless steel crown surfaces. In a scanning electron microscope study conducted by Salama et al.,^16^ concluded that mechanical modifications (sand blasting) create irregular and rough surface with many undercut areas in which the adhesive could wet and penetrate the stainless steel crown's surfaces creating micromechanical retention. In an another similar study done by Grover et al.,^17^ evaluated an *in vitro* effect of sandblasting and laser surface treatment on the shear bond strength of a composite resin to the facial surface of primary anterior stainless steel crowns. They concluded that Nd:YAG laser surface treatment produced an excellent surface roughness and obtained the highest shear bond strength values suggestive for recommendation as an optimal surface treatment to be used to enhance the resin–metal bond at the interface of the composite resin and the facial surface of primary anterior stainless steel crowns for the purpose of chair side veneering.

The most common austenitic steel used in dentistry is the 18-8 stainless steel, so named because it contains approximately 18% chromium and 8% nickel. The carbon content is between 0.08 and 0.20% and titanium, manganese, silicon, molybdenum, niobium, and tantalum are present in minor amount to give important modifications to properties.^18^ The photocatalysis effect by UV irradiation can be a suitable application for dental adhesives as well as composite resins, because the exposure of titanium (Ti) and other materials to UV irradiation induces photoactivation by reducing carbon and increases the oxygen concentrations. Accordingly, diverse metals have been photoactivated, such as chromium (Cr), iron (Fe), and nickel (Ni). UV irradiation over these metals yields a clean and hydrophilic surface through the conversion of carbon to oxygen onto the outer layer. Thus, the UV light on the SSCs induces a photocatalytic effect and improves the metal-bonded resin composite. Baeza-Robleto et al.^19^ used ultraviolet rays with 36 W of power for 80 minutes, he noted that the mean value of shear bond strength was significantly higher for the UV group (19.7 ± 4.3 MPa) than the non-UV group (16.3 + 4.5 MPa).

It has been previously reported that UV irradiation also increases the bonding between composite resin and acrylic resin. Loyaga et al.^20^ in a similar study determined the effect of ultraviolet light irradiation on bonding of experimental composite resin to artificial teeth. Their purpose was to evaluate how UV irradiation using an ordinary UV sterilizer would affect the bonding of experimental composite resins to an auto-polymerizing acrylic resin. Their shear bond strengths after UV irradiation for 1 to 60 minutes were significantly greater than those before UV irradiation regardless of the composite resin type. The failure mode after UV irradiation for 1 to 60 minutes was mainly cohesive failure of the composite resins, but that before UV irradiation and after 24 hours’ irradiation was mainly adhesive failure. These results suggested that a short period of UV irradiation on composite resin teeth would improve the bonding efficacy of composite resin to the auto-polymerizing acrylic resin.

The UV source used in the study is easily available in the dental clinics, in the form of ultraviolet chamber which is commonly used for keeping the surgical instruments. In the study after the exposure of UV rays, a thin layer of metal composite bond (SR linking bonding system) was applied over the crown surface to further improve the retention of composites over the crown. It is based on the phosphoric ester with a methacrylate function. The phosphoric acid group of the molecule is a strong acid, which reacts with the metal or the metal oxides, forming a phosphate. The phosphates form what is known as a passivating layer on the metal surface. After the metal oxide reaction has been completed, the layer becomes very inert. The methacrylate group of the phosphoric acid reacts with the monomer components of SR link, forming a copolymer and, thereby, providing a bond to the veneering resin. Hydrolytic stability (insensitivity to moisture) is achieved, as SR link comprises a monomer that contains a highly hydrophobic aliphatic hydrocarbon chain.

The results of the study showed that the UV group had a statistically significant difference in the mean value of the shear bond strength as compared to the non-UV group. The shear bond strength in the UV was much higher as compared with the non-UV group. This finding was in parallelism with the previous studies reported.

In future studies, a powerful UV lamp could be used to enhance the photocatalytic effect. Further investigations can focus on the optimal time and distance needed to reach quick and total photocatalysis, higher wattages of UV irradiation, and how to achieve optimum attachment of composites by increasing the layers covering the entire surface. The study's findings substantiate that UV-irradiated crowns show advantages over nonirradiated crowns, suggesting their better clinical use.

## CONCLUSION

Based on this study's results, the following conclusion can be made: ultraviolet irradiation of pediatric stainless steel crowns was found to significantly increase the shear bond strength of composite resin.

## CLINICAL SIGNIFICANCE

UV irradiation could provide suitable adhesion of composite resins to stainless SSCs, leading to in-office veneering of SSCs.
